# From brain drain to geopolitical talent competition: scientific diasporas and the future of Global South development

**DOI:** 10.3389/frma.2026.1898634

**Published:** 2026-09-16

**Authors:** Derya Buyuktanir Karacan, Daniel Tapia Takaki

**Affiliations:** 1 The Institute for International Science and Technology (IISPT), Elliott School of International Affairs, George Washington University, Washington, DC, United States; 2 Department of Physics and Astronomy, The University of Kansas, Lawrence, KS, United States

**Keywords:** Global South, networks and ecosystems, scientific diasporas, South–South, science technology and innovation (STI) ecosystems, science diplomacy, network of networks

## Abstract

Scientific diasporas have emerged as increasingly important actors in global science, technology, and innovation (STI) ecosystems. In the context of geopolitical fragmentation, technological competition, and intensifying global inequalities, talent mobility is no longer solely a migration issue but a strategic dimension of development, science diplomacy, and international influence. This policy review argues that Global South countries should move beyond traditional “brain drain” narratives and instead adopt long-term strategies that view scientific diasporas as transnational development partners and components of broader “networks of networks.” Drawing on literature related to scientific diasporas, global talent competition, science diplomacy, and migration studies, this review analyzes how scientific diasporas contribute to development through knowledge transfer, institutional partnerships, capacity building, innovation ecosystems, and policy engagement. It also examines the implications of recent geopolitical shifts, including the growing global competition for talent, technological nationalism, changes in U.S. science and immigration environments, Europe's strategic talent policies, and the rise of emerging scientific hubs beyond the West. Particular attention is given to the Global South, South–South cooperation, reverse brain drain, and the importance of equitable and inclusive knowledge systems. The paper concludes with policy recommendations aimed at strengthening science, technology, and innovation ecosystems and integrating scientific diasporas into national and international development strategies.

## 1 Introduction

Scientific knowledge increasingly shapes geopolitical power, economic resilience, technological sovereignty, and global influence (Crespi et al., 2025; Zegart, 2024). This trend is particularly visible in areas such as artificial intelligence, biotechnology, semiconductors, quantum technologies, climate technologies, and advanced computing, where scientific capacity directly affects national competitiveness and security (National Security Commission on Artificial Intelligence, 2021). Scientific talent has become one of the most strategic resources of the 21st century. In today's knowledge-driven economy, countries compete not only through military strength, trade, and industrial production, but also through their ability to attract, retain, and mobilize highly skilled researchers, engineers, innovators, and entrepreneurs.

The growing importance of knowledge and innovation has intensified global competition for talent. Highly skilled migration has become one of the defining characteristics of globalization. Skilled migration between the Global North and South has emerged as a central component of global economic transformation and innovation systems (Bailey and Mulder, 2017). The increasing mobility of researchers, scientists, and professionals reflects the broader internationalization of higher education, research collaboration, and global labor markets.

However, the globalization of science and talent remains deeply unequal. The Global North continues to dominate research funding, scientific publishing, innovation infrastructure, and higher education rankings, while many Global South countries struggle with underfunded research universities, limited laboratory infrastructure, political instability, weak innovation ecosystems, and restricted career opportunities for researchers (Büyüktınır Karacan and Ruffini, 2023). The 2025 Global Talent Competitiveness Index highlights that global inequalities in talent systems continue to widen, particularly between advanced economies and large parts of Africa, Latin America, and Central Asia (Monteiro et al., 2025). These structural inequalities shape global talent flows and reinforce asymmetrical knowledge systems.

Historically, the migration of scientists and highly educated professionals from the Global South to the Global North was framed through the concept of “brain drain.” Brain drain refers to the loss of educated individuals from developing countries, often trained with public resources, who permanently migrated to wealthier economies offering better salaries, stronger research infrastructure, and greater academic opportunities. This perspective viewed migration largely as a developmental loss that weakened domestic science, technology, and innovation (STI) systems and increased dependency on Northern knowledge centers. Yet contemporary debates increasingly challenge this narrow interpretation. Advances in digital communication, international collaboration, virtual mobility, and transnational networking have transformed how scientists engage with their countries of origin. Scientific diasporas today are not simply expatriate communities disconnected from their homelands. Rather, they often function as transnational knowledge actors who maintain active intellectual, institutional, and emotional ties with their countries of origin while simultaneously participating in global scientific systems.

Scientific diasporas contribute through research collaboration, technology transfer, mentoring, policy advising, entrepreneurship, science diplomacy, and international networking. Tejada and Bolay argue that scientific diasporas should be understood as development partners capable of facilitating knowledge exchange and supporting STI systems in their home countries (Tejada and Bolay, 2010). This perspective shifts the discussion from brain drain toward concepts such as brain circulation, brain linkage, reverse brain drain, and transnational innovation networks.

This paper argues that Global South countries should strategically integrate scientific diasporas into broader development, STI, and science diplomacy agendas. Scientific diasporas should not be viewed merely as symbolic national assets, but as components of broader “networks of networks” that connect universities, laboratories, startups, funding agencies, embassies, international organizations, and digital scientific communities across borders. Such networks can help strengthen domestic research ecosystems, facilitate global collaboration, support innovation, and improve participation of Global South countries in global knowledge governance. At the same time, the paper emphasizes that diaspora engagement alone cannot compensate for structural weaknesses in domestic STI systems. Scientific diasporas are most effective when combined with long-term investments in higher education, research infrastructure, innovation ecosystems, and inclusive governance structures. Without these conditions, talent mobility may continue to reinforce global inequalities rather than reduce them.

From a policy perspective, this policy review argues that Global South countries should move beyond symbolic or short-term diaspora initiatives and instead adopt long-term science diplomacy and STI strategies that integrate scientific diasporas into national development agendas. This requires stronger institutional coordination, investment in domestic research capacity, more equitable international partnerships, and policies that support brain circulation rather than one-directional talent extraction. At the same time, developed countries and international organizations should support more balanced and reciprocal collaboration frameworks that strengthen global scientific cooperation while reducing existing asymmetries in knowledge production, research infrastructure, and innovation capacity.

The purpose of this review is to reassess how Global South countries, developed countries, and international organizations might more strategically engage scientific diasporas, moving beyond a “brain drain” framing toward long-term, network-based approaches to talent mobility, science diplomacy, and development policy. To this end, the review first traces the conceptual shift from brain drain toward brain circulation and “network-of-networks” framings of diaspora engagement, before examining scientific diasporas as networked actors within science diplomacy and Global South development. It then situates this discussion within the intensifying global competition for scientific talent and Europe's expanding role in international scientific collaboration, before turning to the challenges and emerging opportunities facing scientific diasporas. The review concludes by distilling these threads into policy recommendations addressed to Global South countries, developed countries, and international organizations, and by proposing geotalent as a concept warranting further theoretical development.

Consistent with the “Policy and Practice Reviews” article type, this contribution is conceived as a discursive, qualitative policy review rather than a systematic review or an empirical study. It does not report primary data collection; its conclusions rest instead on the interpretive synthesis of the existing academic and policy literature, rather than on a reproducible search protocol such as PRISMA, which is designed for systematic reviews of empirical studies and is not intended for narrative or discursive policy reviews (Baethge et al., 2019). The review accordingly draws on academic literature in science diplomacy, migration studies, innovation systems, and international relations, complemented by institutional and policy reports from bodies such as the European Commission, the OECD, and AAAS, whose data on talent flows, funding environments, and policy design remain, in many cases, the most current available.

Finally, because “the Global South” spans highly heterogeneous national contexts, from large upper-middle-income economies such as India, China, and Brazil to smaller and lower-income states across Sub-Saharan Africa, Central Asia, and the Caribbean, the country examples discussed throughout this review (e.g., Colombia's Caldas Network, India's Silicon Valley diaspora, Mexico's scientific diaspora, and China's “Sea Turtle” returnees) are presented as illustrative cases of particular diaspora-engagement mechanisms rather than as evidence of a single, uniform “Global South” experience. Institutional capacity, political stability, and diaspora size and composition differ substantially across these contexts, and the applicability of any mechanism discussed here should be assessed against these local conditions rather than assumed.

## 2 From brain drain to network-of-networks approaches

The evolution of scientific mobility from the traditional concept of “brain drain” toward “brain circulation,” “brain linkage,” and transnational scientific networking marks one of the more significant shifts in contemporary science, technology, and innovation (STI) policy. For much of the 20th century, the migration of highly skilled professionals from developing to industrialized economies was read as a one-directional loss of human capital, reinforcing asymmetries between the Global North and South (Docquier and Rapoport, 2011). This framing is not merely a historical artifact: recent panel-data analysis of skilled migration across 178 countries between 2006 and 2022 confirms that uneven economic development, weak public services, and external intervention remain robust predictors of high-skilled emigration well into the 21st century, indicating that the structural dynamics first identified in the 20th-century literature have persisted, even as their policy framing has shifted (Vega-Muñoz et al., 2025). Globalization and the growing internationalization of science gradually displaced this reading: migration came to be understood less as a permanent loss than as a potential source of collaboration and knowledge circulation, laying the groundwork for contemporary diaspora and talent policies (Tejada and Bolay, 2010). A similar reassessment runs through Chávez Rodríguez et al. (2026) recent reflection on scientific diasporas, which draws on early-career researchers' own experience to argue that diaspora engagement, left unsupported by governments and institutions, risks becoming dysfunctional and reproducing the very migration it seeks to offset. Unlike recent reflective contributions that focus primarily on the experiences and organization of scientific diasporas (e.g., Chávez Rodríguez et al., 2026), this review situates scientific diasporas within the broader context of geopolitical talent competition, science diplomacy, and STI policy, proposing a “network-of-networks” framework for Global South development.

This framework draws on theories of networked governance developed by Castells (1996), Slaughter (2004), Haas (1992), Gibbons et al. (1994), and Wagner (2018), which describe knowledge and innovation as increasingly circulating through interconnected transnational nodes rather than isolated national institutions. Castells' concept of the “network society” captures how power and knowledge move through global networks rather than remaining confined within borders; Slaughter's account of transgovernmental networks and Haas' concept of epistemic communities help explain how scientific diasporas function not only as scientific actors but also as agents of science diplomacy and global governance.

Central to this transformation is brain circulation: rather than a permanent departure, migration increasingly involves the multidirectional movement of knowledge, expertise, and resources across borders, with diaspora scientists contributing with or without physical return (Commander et al., 2004; Varma and Kapur, 2013; Bonilla et al., 2023). Traditional talent destinations have likewise adapted their own policies to preserve their competitive position in this changing landscape (Kerr et al., 2016).

Reverse brain drain illustrates this shift concretely. China's “Sea Turtles”—returnee scientists and entrepreneurs—helped build globally competitive research institutions as policy moved from urging scientists to “return and serve the motherland” toward more flexible transnational engagement (Tejada and Bolay, 2010; Leslie, 2026); recent funding uncertainty in the United States has only widened this opening. India leveraged its Silicon Valley diaspora to strengthen Bangalore's technology ecosystem (Pandey et al., 2022), while Mexican scientists abroad have sustained strong international research linkages that reinforce, rather than weaken, domestic scientific visibility (Marmolejo-Leyva et al., 2015). South–South collaboration adds a further layer: BRICS countries increasingly draw on shared research infrastructure to reduce dependence on Northern knowledge centers (Echeverría-King et al., 2022), while Dubai, Singapore, India, Malaysia, and Saudi Arabia each pursue distinct strategies—from global talent hubs to diaspora engagement to domestic capacity-building (Ewers et al., 2022).

Innovation-systems literature reinforces this account: innovation emerges through interactive learning among universities, governments, and firms (Lundvall, 1992), within increasingly transnational production and knowledge networks (Ernst, 2002). Spain's RAICEX network, a bottom-up association of Spanish scientists abroad, exemplifies the “network-of-networks” model in practice, combining a diaspora platform with an advisory role in national talent and innovation strategy (Ortega-Paíno and Oliver, 2022); comparable structures are emerging across Latin America, Africa, and Asia.

These developments do not resolve the structural inequalities—weak infrastructure, underfunded universities, limited career pathways—that continue to shape global scientific mobility; diaspora engagement complements, but cannot substitute for, sustained investment in domestic STI capacity.

## 3 Scientific diasporas as networked actors in science diplomacy and Global South development

Science diplomacy has evolved significantly in recent decades alongside the growing interconnection between science, technology, innovation, and global governance as well as the adversarial geopolitics, competitive scientific landscapes, and pressing environmental threats (European Commission, 2025; AAAS and The Royal Society, 2025). The COVID-19 pandemic intensified global competition for skilled talent, while growing recognition of the importance of science-informed policymaking encouraged many nations to embed scientific expertise within foreign ministries (Ruffini, 2022). Switzerland's Swissnex, Germany's DWIH, and the United Kingdom's Science and Technology Network (STN, formerly SIN) exemplify developed country-specific institutional models. It has also expanded its reach to developing and emerging countries. Countries in the Global South and developing countries, including Brazil, India, Pakistan, Colombia, and Malaysia, have increasingly adopted science diplomacy as both a tool for global engagement and a mechanism for development (Büyüktınır Karacan, 2021; Echeverría-King et al., 2021). Rather than primarily emphasizing soft power, these countries often view science diplomacy to strengthen local capacities, support innovation, and enhance participation in global research networks. This evolution has also renewed debates on inequality and asymmetry in international scientific partnerships. Importantly, Global South countries bring diverse priorities and perspectives to science diplomacy, challenging traditional paradigms and broadening the scope of international scientific cooperation. Nevertheless, reconciling national interests with global collaboration remains a persistent challenge in both developed and developing contexts.

As Section 2 outlined, Castells (1996), Slaughter (2004), and Haas (1992) each describe knowledge and governance as increasingly organized through transnational networks rather than confined within national institutions. Within this architecture, scientific diasporas function as non-state actors linking local and global knowledge systems: operating simultaneously across multiple institutional, political, and cultural settings, they can be understood—following Haas (1992)—as transnational epistemic communities that contribute to development-oriented science diplomacy as much as to scientific collaboration itself.

The growing importance of scientific diasporas is also closely connected to broader transformations in knowledge production and innovation systems. Gibbons et al. (1994) argue that knowledge production has become increasingly transdisciplinary, socially distributed, and application-oriented, moving beyond isolated academic institutions toward globally interconnected systems of collaboration. Nowotny et al. (2001) further emphasize that science is increasingly produced within complex contexts shaped by uncertainty, globalization, and societal demands. While these accounts are now three decades old, the trend they describe has, if anything, intensified with digitalization and AI-driven research, even as its specific mechanisms have evolved considerably. These changes have important implications for Global South countries, where scientific diasporas can help connect domestic institutions to international research ecosystems, funding opportunities, and innovation networks. Lundvall's work on national systems of innovation demonstrates that innovation emerges through interactive learning among universities, governments, firms, and broader social actors, while Ernst's research on global production and innovation networks highlights the increasingly transnational character of technological development and knowledge flows (Ernst, 2002; Lundvall, 1992). Scientific diasporas frequently operate across these interconnected systems by facilitating technology transfer, collaborative research, entrepreneurship, mentoring, and institutional partnerships. In many developing countries, diaspora scientists also help compensate for structural limitations in research infrastructure, international visibility, and access to international funding.

The “network of networks” perspective reframes migration beyond traditional “brain drain” narratives. Meyer and Brown (1999) frame this network logic as an alternative to brain drain, in which diaspora scientists promote circulation and collaborative capacity building rather than permanent departure—a pattern Tejada and Bolay (2010) confirm empirically across the Colombian, Indian, and South African cases discussed above. Through such networks, diaspora communities support research collaboration, policy advice, entrepreneurship, and institutional capacity building, while also strengthening South–South and North–South cooperation.

Scientific diasporas also play several strategic roles within this evolving science diplomacy architecture. Their contributions go far beyond traditional understandings of migration and increasingly position diaspora communities as active actors within global knowledge systems, innovation ecosystems, and development strategies. These contributions can be understood across several interconnected dimensions:

*Capacity building*: scientific diasporas contribute significantly to strengthening higher education and research capacity in their countries of origin. Diaspora scientists frequently support the development of postgraduate programs, advanced scientific training, joint supervision mechanisms, curriculum development, and institutional partnerships between universities and research centers (Bonilla et al., 2023; Tejada and Bolay, 2010; Avendano-Uribe et al., 2022). In countries where research institutions face limited funding, weak infrastructure, or insufficient international visibility, these forms of transnational engagement can help strengthen local scientific ecosystems, improve human capital development, and facilitate access to global research communities. They serve as scientific bridge between other countries and the host country (Burns, 2013).

*Technology and knowledge transfer*: diaspora scientists often function as important conduits for advanced technologies, scientific expertise, innovation practices, and international funding opportunities. Their transnational professional networks can facilitate collaborative research projects, startup ecosystems, entrepreneurship, and technology transfer, particularly in sectors such as information and communication technologies (ICT), biotechnology, renewable energy, public health, and artificial intelligence (Bailey and Mulder, 2017; Tejada and Bolay, 2010). Through these activities, scientific diasporas can help integrate developing countries into broader global innovation systems and emerging technology ecosystems.

*Trust-building and mediation*: because diaspora scientists frequently maintain professional, institutional, and cultural ties across multiple countries, they can also play important diplomatic and mediating roles. Scientific diaspora networks may provide informal “listening channels” between governments, universities, and scientific communities, particularly during periods of geopolitical tension or strained diplomatic relations (Brinkerhoff, 2019). In some cases, diaspora communities can act as relatively neutral intermediaries that sustain scientific collaboration and dialogue even when formal diplomatic channels become more limited. Diasporas possess a unique “in-between advantage” that allows them to connect societies and policy communities across borders in ways that traditional institutions sometimes cannot.

*Policy advice and evidence-based governance*: highly skilled professionals residing abroad are increasingly incorporated into national science advice mechanisms, expert committees, advisory boards, and strategic STI policy discussions. Their international experience and exposure to multiple research and innovation systems can provide valuable comparative perspectives for national development strategies, innovation governance, and science diplomacy frameworks. In an era characterized by global technological competition, climate challenges, digital transformation, and geopolitical fragmentation, diaspora scientists are therefore becoming increasingly strategic actors not only within scientific collaboration, but also within evidence-based policymaking and international science diplomacy processes.

## 4 Global competition for talent

Global competition for scientific talent has intensified significantly in recent years as countries increasingly recognize highly skilled researchers, innovators, and entrepreneurs as strategic assets linked to economic competitiveness, technological sovereignty, and geopolitical influence. Highly skilled migration is no longer viewed solely as a labor-market issue but also as a central component of national innovation systems and science diplomacy strategies. Highly skilled migration between the Global North and South has become deeply connected to broader transformations in global economic systems, knowledge production, and international labor markets (Bailey and Mulder, 2017). Similarly, as Docquier and Rapoport (2011) emphasize that globalization has increased the concentration of scientific talent within countries with stronger innovation ecosystems and research infrastructures, thereby reinforcing existing asymmetries in global STI systems.

The United States historically dominated global talent attraction through its universities, venture capital ecosystem, scientific infrastructure, and relatively open research environment. Immigrant scientists and researchers continue to play a disproportionately important role within the U.S. innovation system and research workforce (Kerr, 2020; Neufeld and Kaushik, 2024). However, recent geopolitical and domestic developments have raised concerns about the long-term attractiveness of the United States to international talent. Restrictive immigration debates, significant reductions in federal research funding, the restructuring of federal funding agencies, declining support for some research universities, growing research security concerns, and increasing political polarization risk undermining the United States' long-standing competitive advantage in attracting global researchers and innovators (Teich, 2014; Stephan et al., 2016; Burrows and Hardegree, 2026). Similar concerns are also reflected in discussions of technological competition, particularly regarding China, semiconductors, artificial intelligence, and strategic research sectors.

Recent global talent data indicate important shifts in international competition for highly skilled researchers, innovators, and entrepreneurs. According to the Global Talent Competitiveness Index (GTCI) 2025, Singapore surpassed Switzerland to become the world's leading talent economy, reflecting the growing importance of Asian innovation ecosystems and strategic talent policies (Monteiro et al., 2025). The report highlights Singapore's strong performance in areas such as regulatory environment, adaptability, digital readiness, and talent attraction, demonstrating how smaller states can position themselves as major global knowledge hubs through long-term investment in education, innovation, and international openness. At the same time, the United States declined from 3rd place in the 2023 rankings to 9th place in the GTCI 2025 rankings, reflecting concerns related to labor market conditions, institutional uncertainty, and broader business climate challenges, despite the country's continued strength in higher education, research infrastructure, and innovation capacity (Monteiro et al., 2025).

Europe nevertheless continues to dominate many dimensions of global talent competitiveness, with countries such as Switzerland, Germany, France, and the Nordic states maintaining strong overall performance (Monteiro et al., 2025).

At the same time, OECD indicators on talent attractiveness present a somewhat different but complementary perspective on global talent mobility. According to the OECD's (2023) Indicators of Talent Attractiveness, countries such as Australia, Sweden, Switzerland, New Zealand, and Canada consistently rank among the most attractive destinations for highly qualified workers and entrepreneurs because of favorable labor markets, strong skills environments, quality of life, and supportive business conditions. The OECD analysis also emphasizes that immigration barriers, high international tuition fees, administrative backlogs, and restrictive visa environments negatively affect the attractiveness of several advanced economies. These findings suggest that global competition for talent increasingly depends not only on scientific excellence and economic performance, but also on broader governance environments, mobility systems, openness, and long-term integration policies.

As all these reports and growing body of evidence demonstrate global talent competition is becoming increasingly multidirectional and dynamic. The emergence of countries such as Singapore, South Korea, the United Arab Emirates, and Canada as highly competitive talent destinations indicates that scientific and technological leadership is no longer concentrated exclusively within traditional Western powers. At the same time, these global rankings continue to reveal major asymmetries between advanced economies and many countries in Africa, Latin America, and parts of Asia, where structural limitations in higher education systems, research infrastructure, innovation ecosystems, and funding capacity continue to constrain talent development and retention.

## 5 Europe, international scientific collaboration, and the expansion of “network-of-networks” approaches

The European Union has become one of the most active global actors supporting transnational scientific collaboration, scientific mobility, and “open to the world” approaches through its science diplomacy, research, and innovation frameworks. Over the past decade, the EU has increasingly positioned science, technology, and innovation (STI) as strategic components of both competitiveness and international cooperation through various initiatives such as Horizon Europe Research and Innovation Program [including the Marie Skłodowska-Curie Actions (MSCA), European Research Council (ERC) Grants], collaborative projects, COST Actions, Erasmus+, Choose Europe for Science, and the Global Gateway strategy. The European Union's Global Approach to Research and Innovation (European Commission, 2021) promotes science and technology as tools for shaping norms and extending soft power across international partnerships. It has expanded collaboration with researchers, institutions, and scientific diasporas across globe including Africa, Latin America and the Caribbean (LAC), ASEAN countries, and other regions of the Global South (European Commission, 2022).

Horizon Europe is no longer simply a European funding program. It is becoming a global platform for research collaboration and a central pillar of the EU's Global Approach to Research and Innovation (Linthorst, 2026). In 5 years', 14 non-EU countries have joined, including South Korea, Egypt, New Zealand and Japan. In March 2026, talks were opened with Australia. The program increasingly functions as a mechanism for projecting soft power, fostering international partnerships, positioning the EU as a leading global actor in science, technology, and innovation, and attracting highly skilled talent. These initiatives increasingly support multidirectional scientific mobility, transnational knowledge exchange, institutional partnerships, and collaborative innovation ecosystems rather than traditional one-directional North–South cooperation models.

One of the clearest examples of this “network-of-networks” model is the European Cooperation in Science and Technology (COST) program (COST, 2026). COST Actions function as open and flexible international research networks connecting researchers, universities, policymakers, early-career researchers, industry actors, and civil society organizations across Europe and beyond since 1971. Unlike traditional research grants focused primarily on scientific outputs, COST, as model of networking, emphasizes networking, coordination, interdisciplinary collaboration, training, and long-term community building (Kostelidou and Babiloni, 2010). Recent COST activities increasingly integrate science diplomacy, co-production of knowledge, policy engagement, and international collaboration with Global South partners.

The contemporary organization of frontier science increasingly draws on distributed “network-of-networks” (NoN) governance architectures to coordinate across disciplines, institutions, and geopolitical boundaries. This is not merely an aspirational label: a mixed-methods evaluation of NoN arrangements at the science-policy interface finds that mobilizing existing networks through a NoN structure can improve coordination and capacity-building, while also cautioning that its viability depends on participating networks sharing sufficiently aligned incentives and on sustained attention to institutional memory and coordination costs (Kelemen et al., 2021). We therefore treat the NoN framing here as one increasingly employed, rather than an uncontested or default, architecture for organizing transnational scientific collaboration.

In this regard, the U.S. National Science Foundation (NSF)'s Accelerating Research through International Network-to-Network Collaborations (AccelNet) program represents an important and forward-looking model for the future of global scientific collaboration in the United States (National Science Foundation, 2023). Rather than prioritizing isolated projects or institution-centered competition, AccelNet emphasizes the development of interoperable international research ecosystems capable of sustaining long-term collaboration, scientific capacity building, and globally connected innovation communities. By leveraging prior investments in research capacity and fostering internationally competent scientific leadership, the program recognizes that contemporary scientific challenges require transnational coordination infrastructures rather than fragmented national efforts alone. As mentioned above, the European COST framework represents another important example of this approach, and the growing cooperation between NSF AccelNet and EU COST initiatives reflects the inherently global nature of contemporary scientific questions and the transnational character of today's grand challenges. However, recent political and financial changes in the United States have created uncertainty regarding the long-term sustainability of some international collaboration initiatives. The reduction of federal science funding and broader budgetary pressures associated with recent U.S. political developments, including cuts affecting the National Science Foundation (NSF), have raised concerns about the future of large-scale international networking programs such as AccelNet. Importantly, these models do not only benefit internationally mobile researchers or scientific diasporas, but also provide critical training environments for domestic students and early-career researchers, who increasingly require experience working within multidisciplinary, multicultural, and geographically distributed teams in both globalized and increasingly fragmented scientific and technological ecosystems. Expanding participation in such collaborative models across STEM disciplines can significantly strengthen workforce preparation, scientific adaptability, and international scientific competence. It is precisely through these distributed frameworks that a more effective leverage of scientific resources, expertise, and infrastructures becomes possible, while scientific diasporas serve as connectors, coordinators, and catalysts for the development of globally integrated scientific communities. Within this emerging landscape, scientific diasporas evolve from passive communities abroad into active infrastructures of coordination that connect institutions, researchers, and innovation ecosystems across borders.

The international collaborative ecosystem surrounding The European Organization for Nuclear Research, (CERN) provides one of the clearest examples of how large-scale scientific infrastructures can catalyze the formation and consolidation of globally interconnected scientific diaspora networks. Beyond its role as a leading research facility in high energy physics, CERN has developed into a transnational hub of scientific exchange that connects researchers, students, engineers, and institutions through conferences, workshops, mobility programs, training initiatives, collaborative experiments, and distributed research networks. Such ecosystems are critical because scientific communities do not emerge spontaneously; they require stable institutional hubs capable of concentrating talent, facilitating repeated interaction, and sustaining long-term collaboration across generations of researchers. In this sense, scientific diaspora hubs may play a strategic role in strengthening national and regional scientific leadership by serving as focal points for attraction, coordination, and retention of globally mobile talent. Reinterpreting the six imperatives identified by the American Academy of Arts Sciences (2020) (global scientific questions, competition for talent, economic competitiveness, national security, funding realities, and ethical governance) through the lens of scientific diasporas reveals a broader transformation in the structure of international scientific collaboration. These axes underscore the importance of “network of network approach” in fostering international partnerships and advancing scientific knowledge (Crew, 2019). Scientific diasporas increasingly operate as strategic actors embedded within transnational STI systems, simultaneously supporting innovation, scientific diplomacy, and distributed problem-solving while also existing within a context of growing geopolitical fragmentation, technological competition, export controls, and security concerns. As scientific openness becomes increasingly entangled with geopolitical rivalry, scientific diaspora network of networks occupy an ambivalent but central position within the emerging multipolar organization of global science, highlighting the need for promoting new frameworks of scientific diaspora structures capable of balancing collaboration, resilience, technological sovereignty, and international cooperation.

Europe is increasingly integrating international scientific collaboration into broader geopolitical, economic, and talent-attraction strategies. The European Commission's policy framework on legal migration and talent attraction explicitly recognizes that global competition for highly skilled workers is becoming central to Europe's economic recovery, digital transformation, and technological sovereignty agenda. The Commission states that legal migration is essential for addressing long-term skills shortages, supporting innovation ecosystems, and strengthening Europe's competitiveness within emerging technological sectors (European Commission, 2022). European initiatives such as the EU Talent Pool, EURAXESS, and EU Visa Strategy (European Commission, 2026) aim to make the EU more attractive for international researchers, innovators, entrepreneurs, and highly skilled professionals. These developments demonstrate that Europe is increasingly approaching scientific mobility and international collaboration not only through traditional development cooperation but also as part of a broader strategic competition for global talent and innovation leadership.

Europe's growing engagement with Africa, Latin America and the Caribbean (LAC), and ASEAN countries further reflects this broader strategy. Through Horizon Europe association agreements, Global Gateway initiatives, AU-EU research partnerships, and regional science diplomacy frameworks, the EU increasingly supports collaborative STI ecosystems, mobility schemes, research infrastructures, and capacity-building programs across the Global South (European Commission, 2022). The European Commission's report Recommendations on How to Make R&I a Driver for Sustainable Development in AU-EU Relations emphasizes that research and innovation should become central pillars of sustainable development and more equitable international cooperation between Africa and Europe (European Commission, 2022). Similarly, EU-LAC and EU-ASEAN cooperation frameworks increasingly promote joint research agendas, innovation partnerships, digital transformation, climate collaboration, and scientific mobility. Moreover, an EU-funded projects, European Union Global Diaspora Facility (EUDiF) aim at enhancing the engagement and collaboration between diaspora organizations in Europe and their countries of origin for development purposes. These initiatives help strengthen transnational “network-of-networks” structures by connecting universities, scientific diasporas, research infrastructures, policymakers, and innovation actors across regions.

Nevertheless, important asymmetries remain within these international collaboration frameworks. European institutions continue to possess stronger funding capacities, research infrastructures, and institutional coordination mechanisms than many Global South partners. Participation in large European research programs often requires substantial administrative capacity, grant management expertise, digital infrastructure, and international visibility, which may disadvantage less-resourced institutions. Scholars of science diplomacy and decolonial knowledge systems therefore increasingly emphasize the importance of moving beyond extractive or hierarchical partnership models toward more equitable forms of co-production, shared leadership, and reciprocal scientific collaboration (Polejack et al., 2022; Büyüktınır Karacan and Ruffini, 2023). Despite these challenges, Europe's expanding support for international scientific networking, talent mobility, and transnational collaboration frameworks demonstrates how collaborative approaches are becoming central components of contemporary science diplomacy and global STI governance.

## 6 Challenges and emerging opportunities

Scientific diasporas increasingly play important roles within global science, technology, and innovation (STI) systems, particularly in relation to Global South development, science diplomacy, and transnational knowledge exchange. However, their long-term impact depends on broader political, institutional, and geopolitical conditions that can either constrain or strengthen diaspora engagement. Recent scholarship therefore examines scientific diasporas not only through their developmental contributions, but also through the challenges and emerging opportunities shaping their role within global knowledge systems (Polejack et al., 2022; AAAS and The Royal Society, 2025; Bonilla et al., 2023).

Despite growing international attention to diaspora engagement, several structural and geopolitical challenges continue to limit the effectiveness and sustainability of scientific diaspora initiatives.

*Political and institutional instability*: many Global South countries continue to face governance challenges, inconsistent STI policies, limited research funding, and institutional instability. Diaspora initiatives are often dependent on temporary political support, short-term projects, or individual leadership rather than long-term national strategies (Bonilla et al., 2023). As a result, many collaboration mechanisms become fragmented or difficult to sustain over time.

*Restrictive mobility and geopolitical tensions*: increasing geopolitical competition surrounding emerging technologies, research security, migration control, and technological sovereignty increasingly affects scientific mobility and international collaboration. Visa restrictions, administrative barriers, and geopolitical tensions may reduce opportunities for researchers from developing countries to participate fully within global scientific ecosystems. This is particularly visible in strategic sectors such as artificial intelligence, semiconductors, biotechnology, and cybersecurity.

*Persistent global knowledge asymmetries*: scientific diasporas continue to operate within highly unequal global knowledge systems. Researchers and institutions in many developing countries face structural disadvantages related to research infrastructure, publication visibility, access to funding, digital capacity, and participation in international agenda-setting processes. International scientific collaboration may unintentionally reproduce existing inequalities if partnerships are not designed in more equitable and reciprocal ways.

*Coordination and sustainability challenges*: many scientific diaspora networks remain volunteer-driven and dependent on informal structures or temporary funding schemes. Maintaining long-term engagement, institutional memory, stable governance, and strategic coordination therefore becomes difficult over time. The sustainability of networks requires stronger institutional support and long-term policy planning.

### 6.1 Emerging opportunities and new global dynamics

At the same time, several recent developments create important opportunities for scientific diasporas and Global South development.

*Digital scientific collaboration and virtual networks*: the rapid expansion of virtual laboratories, online research platforms, AI-supported networking tools, and open science infrastructures increasingly allows diaspora scientists to contribute without permanent physical return. These digital systems help facilitate collaborative research, mentoring, policy dialogue, and scientific exchange across borders.

*Growing international attention to science diplomacy*: recent international discussions increasingly emphasize inclusive science diplomacy, equitable collaboration, and stronger Global South participation within STI governance systems (AAAS and The Royal Society, 2025; European Framework for Science Diplomacy, 2025). This creates opportunities for diaspora scientists to become more actively involved in international scientific cooperation and policy dialogue.

*Expansion of regional and interregional collaboration*: increasing number of scientific partnerships with Global South countries increasingly support mobility schemes, collaborative research, innovation ecosystems, and scientific networking. Programs such as Horizon Europe, COST Actions, and Global Gateway initiatives contribute to broader “network-of-networks” approaches that connect universities, scientific diasporas, policymakers, and innovation actors across regions.

*Growth of South–South scientific cooperation*: countries such as India, China, Brazil, South Africa, Singapore, and the Gulf states increasingly function as emerging scientific and technological hubs. These developments create new opportunities for South–South collaboration, multidirectional mobility, and regional innovation partnerships that are not exclusively dependent on traditional Western scientific centers.

Within this evolving environment, scientific diasporas contribute strategically to development, science diplomacy, and global knowledge systems in several ways as discussed in the previous sections.

## 7 Policy recommendations

The growing competition for scientific talent, the rise of emerging technologies, and the widening asymmetries in global STI capacity require more balanced and realistic policy responses. Scientific diasporas should not be treated only as a solution to brain drain, nor should they be expected to compensate for weak domestic research systems. Rather, they should be integrated into broader science, technology, innovation, development, and science diplomacy strategies. The following recommendations distinguish between short-term and long-term actions for Global South countries, developed countries, and international or regional organizations.

### 7.1 Recommendations for Global South countries

In the short term, Global South governments should begin by systematically mapping their scientific diasporas. Many countries lack reliable data on where their researchers are located, what expertise they possess, or how willing they are to contribute. Digital platforms, publication databases, professional networks, and, where appropriate, AI-supported tools can help identify diaspora expertise and connect it with national priorities. However, such mapping should be transparent, voluntary, and designed to build trust rather than control. This responds to the need for more organized diaspora engagement highlighted in studies of Latin American and Caribbean scientific diasporas and in the Guatemala and Mexico cases (Echeverría-King et al., 2022; Bonilla et al., 2022; Gómez-Flores et al., 2022).

Global South countries should also create practical “brain linkage” mechanisms rather than focusing only on permanent return. Many diaspora scientists may not be able or willing to return permanently, but they can still contribute through joint research, virtual mentoring, co-supervision of graduate students, short-term visits, policy advice, and collaborative grant applications. This approach builds on the shift from brain drain to brain circulation emphasized by Meyer and Brown (1999) and Tejada and Bolay (2010). Small seed grants for diaspora-local research teams, online advisory groups, and diaspora lecture series could create immediate impact without requiring large budgets. This institutional and policy-level framing complements, rather than replaces, the bottom-up, personal-choice-driven perspective on diaspora engagement recently articulated by Chávez Rodríguez et al. (2026), whose reflections on early-career mobility speak to the individual experiences that institutional mechanisms such as these are meant to support.

In the medium and long term, Global South countries need to institutionalize scientific diaspora engagement within national STI and foreign policy systems. This could include dedicated diaspora units within science ministries, science diplomacy desks in foreign ministries, and science attachés in embassies located in major research hubs such as Boston, London, Berlin, Paris, Toronto, Singapore, and Silicon Valley. Colombia's Caldas Network—discontinued after connecting 29 nodes of expatriate scientists, and now a widely cited case of how diaspora networks can fragment once nodes specialize faster than coordination can keep pace (Echeverría-King, 2022)—alongside Mexico's Red Global MX, Argentina's RAICES program, Costa Rica's Hipatia platform, and India's Ramanujan Fellowship, illustrates both the potential and the fragility of such initiatives, which require sustained funding and coordination (Tejada and Bolay, 2010; Echeverría-King et al., 2022; Pandey et al., 2022).

Long-term policy must also focus on domestic absorptive capacity. Scientific diasporas can support development only if home institutions have the capacity to receive, use, and sustain knowledge flows. This means investing in universities, laboratories, research management offices, digital infrastructure, competitive grant systems, and career pathways for early-career researchers. Without these investments, diaspora engagement risks remaining symbolic or dependent on individual goodwill. The goal should not only be to bring scientists back, but to make return, circulation, and collaboration meaningful.

### 7.2 Recommendations for developed countries

Developed countries should recognize that talent-attraction policies can unintentionally deepen global inequalities if designed solely to extract highly skilled researchers from weaker systems. Many developed countries including the United States, Canada, the United Kingdom, Switzerland, Australia, and EU member states all benefit from international scientific mobility. At the same time, they should design policies that support circulation rather than one-way migration. This is especially important as global competition for talent intensifies and countries revise immigration, visa, and research funding policies (Teich, 2014; Kaushik and Watney, 2021; OECD, 2023; Neufeld and Kaushik, 2024).

In the short term, developed countries should make research mobility more predictable and less burdensome. Visa uncertainty, long processing times, high fees, and unclear pathways to residency can weaken international collaboration and discourage talented researchers. At the same time, mobility policies should include mechanisms that strengthen partnerships with countries of origin. For example, postdoctoral fellowships, doctoral networks, and visiting researcher schemes could include optional components for co-supervision, return visits, or joint institutional collaboration with Global South universities.

Developed countries should also support fairer research partnerships. Funding agencies should encourage co-designed projects, shared leadership, equitable authorship, and meaningful capacity-building. Global South partners should not be treated merely as data providers or field sites. Tools such as the Research Fairness Index and the AU-EU recommendations on research and innovation cooperation can help move partnerships toward more balanced models. This is particularly important in areas such as climate, public health, biodiversity, AI, agriculture, and energy, where Global South countries often provide critical knowledge, data, and local expertise.

In the long term, developed countries should align talent attraction with “responsible science diplomacy.” Horizon Europe Program on Research and Innovation can offer important opportunities for international collaboration, but they should be implemented in ways that reduce asymmetries rather than reinforce them. Similarly, research institutions of the developed countries should strengthen international partnerships that support capacity-building abroad while continuing to welcome global talent. The aim should be mutual knowledge circulation, not talent extraction.

### 7.3 Recommendations for international and regional organizations

International and regional organizations can play a major role in connecting fragmented diaspora initiatives and reducing duplication. UNESCO, OECD, the African Union, the European Union, ASEAN, OAS, BRICS, regional development banks and other international organizations could support regional “networks of networks” that connect scientific diasporas across countries and regions. This is especially useful for countries with small scientific diasporas that may not have the scale to build strong national networks alone.

In the short term, these organizations could develop shared platforms for diaspora mapping, the exchange of good practices, and policy learning. They could also support regional training programs for science diplomacy, research management, and international grant writing. Such initiatives would help Global South institutions participate more effectively in global research programs.

In the long term, international organizations should support equitable digital research infrastructure, including virtual laboratories, open data hubs, regional research repositories, and collaborative platforms. These tools can reduce the importance of geography and allow diaspora scientists to contribute from wherever they are based. They are particularly important for countries affected by conflict, climate vulnerability, or limited research infrastructure.

### 7.4 Cross-cutting recommendation

Across all country groups, the key principle should be balance. Scientific diaspora policy should balance mobility and retention, national interest and global cooperation, talent attraction and capacity-building, research security and openness, and excellence and equity. The most effective approach is not to stop migration, nor to rely entirely on return migration, but to build systems that keep knowledge, people, and institutions connected across borders. This is the core value of the “network of networks” approach: it treats scientific diasporas as part of a wider transnational ecosystem that can support development, science diplomacy, and more inclusive global knowledge governance. It is also worth noting that many of the pressures described throughout this paper—the pull toward emigration, the difficulty of sustaining diaspora engagement, the premium placed on international publication and citation counts—are symptomatic of a narrower set of incentives that structure how scientific merit itself is defined and rewarded; addressing brain drain durably may therefore also require rethinking these broader evaluative norms, not only diaspora policy in isolation.

## 8 Summary

This paper has argued that the traditional “brain drain” framework no longer captures the multidirectional, networked character of contemporary scientific mobility. The reconfiguration of the global science, technology, and innovation landscape calls for a rethinking of how highly skilled talent is understood and engaged. This paper proposes the term geotalent to describe highly skilled professionals, researchers, international students, entrepreneurs, and members of scientific diasporas whose mobility and transnational networks are increasingly shaped by geopolitical, technological, economic, and societal change. Amid strategic competition and shifting centers of scientific excellence, these individuals function not merely as participants in global knowledge systems but as key actors at the intersection of science, innovation, diplomacy, and international relations. Recognizing, attracting, retaining, and mobilizing geotalent should therefore become a strategic priority for governments, universities, and research institutions worldwide.

The prefix geo in geotalent is deliberately chosen to signal the multiple registers in which this mobility now unfolds: geography, in the literal sense of shifting routes, destinations, and centers of scientific activity; geopolitics, as talent flows are increasingly shaped by strategic rivalry, alliance structures, and national security considerations; geo-economics, as innovation ecosystems compete for human capital much as they compete for capital and technology; and geo-environment, as climate change and environmental disruption emerge as additional drivers of scientific displacement and relocation. This layered meaning distinguishes geotalent from earlier vocabulary such as brain drain, brain circulation, or skilled migration, which largely describe movement between origin and destination countries without capturing the multidimensional forces now at work. The present paper, centered on the concept of a network-of-networks linking scientific diasporas, research institutions, and policy actors, introduces geotalent as an analytical foundation rather than a fully developed framework; a more extensive theorization of the concept, including its geographic, geopolitical, and geo-environmental dimensions, is intended for forthcoming work by the authors.
